# Exogenous C2 Ceramide Suppresses Matrix Metalloproteinase Gene Expression by Inhibiting ROS Production and MAPK Signaling Pathways in PMA-Stimulated Human Astroglioma Cells

**DOI:** 10.3390/ijms17040477

**Published:** 2016-03-31

**Authors:** Ji-Sun Jung, Young-Ho Ahn, Byung-In Moon, Hee-Sun Kim

**Affiliations:** 1 Department of Molecular Medicine and Tissue Injury Defense Research Center, Ewha Womans University Medical School, Seoul 07985, Korea; kurum77@naver.com (J.-S.J.); yahn@ewha.ac.kr (Y.-H.A.); 2 Department of Surgery, Ewha Womans University Medical School, Seoul 07985, Korea; mbit@ewha.ac.kr

**Keywords:** C2 ceramide, astroglioma, invasion, MMP, signaling mechanism

## Abstract

Matrix metalloproteinases (MMPs) are a family of zinc-dependent endopeptidases, which play a pivotal role in invasion, migration, and angiogenesis of glioma. Therefore, controlling MMPs is potentially an important therapeutic strategy for glioma. In the present study, we found that exogenous cell-permeable short-chain C2 ceramide inhibits phorbol myristate acetate (PMA)-induced *MMP-1*, *-3*, and *-9* gene expressions in U87MG and U373MG human astroglioma cells. In addition, C2 ceramide inhibited the protein secretion and enzymatic activities of MMP-1, -3, and -9. The Matrigel invasion assay and wound healing assay showed that C2 ceramide suppresses the *in vitro* invasion and migration of glioma cells, which appears to be involved in strong inhibition of MMPs by C2 ceramide. Subsequent mechanistic studies revealed that C2 ceramide inhibits PMA-induced mitogen-activated protein kinase (MAPK) phosphorylation and nuclear factor (NF)-κB/activator protein (AP)-1 DNA binding activities. Furthermore, C2 ceramide significantly inhibited PMA-induced reactive oxygen species (ROS) production and NADPH oxidase 4 (NOX4) expression, and inhibition of ROS by diphenylene iodonium (DPI, NADPH oxidase inhibitor) mimicked the effects of C2 ceramide on MMP expression and NF-κB/AP-1 via inhibition of p38 MAPK. The results suggest C2 ceramide inhibits MMP expression and glioma invasion, at least partly, by modulating ROS-p38 MAPK signaling axis and other MAPK signaling pathways.

## 1. Introduction

Matrix metalloproteinases (MMPs) are zinc-dependent endopeptidases able to degrade or remodel extracellular matrix (ECM) proteins in various physiological and pathological conditions [1,2]. MMPs are also involved in cell death, proliferation, differentiation, migration, and cell signaling, thereby contributing to angiogenesis, organogenesis, and wound healing [3]. Malignant gliomas are the most common primary brain tumors in adults, and are characterized by cell proliferation, angiogenesis, and insidious infiltration to the brain [4]. In human gliomas, increased MMP levels promote tumor-cell invasion by degrading the extracellular matrix (ECM) and tightening junction proteins [5]. Therefore, limiting the invasion of tumor cells within a normal brain is one of the major therapeutic targets when it comes to gliomas. It was previously reported that the MMP-1 protein level increases with the tumor grade and is related to increased glioma invasiveness [6]. In addition, MMP-3 plays a critical role in glioma invasiveness through degradation of hyaluronic acid-rich matrix of the brain [7]. MMP-9 is another well-characterized enzyme and has been prominently implicated in glioma invasiveness. The level of MMP-9 was found to be increased during the growth of glioblastoma cells intracerebrally implanted in nude mice [8]. Based on these findings, controlling MMP expression has been proposed as an important therapeutic target for malignant glioma treatments.

Ceramide is one of the central molecules of sphingolipid metabolism and plays a critical role in the regulation of various cellular functions, including cell proliferation, differentiation, migration, and senescence [9]. Previous studies have reported on the role of ceramide in cancer progression. Some studies reported that ceramide contributes to tumor suppressive and anti-proliferative cellular programs, including autophagy, apoptosis, and necroptosis [9,10,11]. Other works reported that ceramides up-regulate or down-regulate the progression of human breast and colon cancer cells depending on the length of side chains [12]. In fact, an increase of long chain ceramides (C_16:0_-, C_18:0_-, and C_20:0_-Cer) is accompanied by an induction of apopotosis of cancer cells, whereas up-regulation of very long chain ceramides (C_24:0_-, C_24:1_-Cer) promotes cell proliferation. These reports suggested that the disequilibrium between long and very long chain ceramides may determine the fate of cells. On the other hand, the cell-permeable short chain C6 ceramide enhanced pemetrexed-induced apoptosis and cytotoxicity in osteosarcoma cells through inhibition of Akt-mammalian target of rapamycin (mTOR) signaling [13]. Moreover, C6 ceramide intensified cytotoxic effects of Akt inhibitor perifosine in glioblastoma cells [14]. A recent study described how C2 ceramide inhibits the invasiveness of human bronchocarcinoma by inhibiting MMP-2 expression [15]. The tumor suppressive function and molecular action of ceramides appear to be fluctuating depending on the stimulus and cell types.

Although a number of studies have reported on the pharmacological activities of ceramide, the effect of C2 ceramide in glioma invasion has not yet been demonstrated. In the present study we, thus, investigated whether C2 ceramide inhibits the expression of MMPs, which play a crucial role in glioma invasion and progression. We discovered that C2 ceramide strongly inhibits the expression and enzymatic activities of MMP-1, -3, and -9 induced by phorbol myristate acetate (PMA) in human astroglioma cells. In addition, C2 ceramide also appears to inhibit the glioma invasion and migration. Further mechanistic studies showed that reactive oxygen species (ROS) and mitogen-activated protein kinase (MAPK) signaling pathways are involved in MMP modulation by C2 ceramide in PMA-stimulated glioma cells. The data collectively suggest a therapeutic potential of C2 ceramide for malignant glioma.

## 2. Results

### 2.1. C2 Ceramide Suppresses the mRNA Levels and Promoter Activities of MMP-1, -3, and -9 in U87MG Glioma Cells

**Figure 1:**
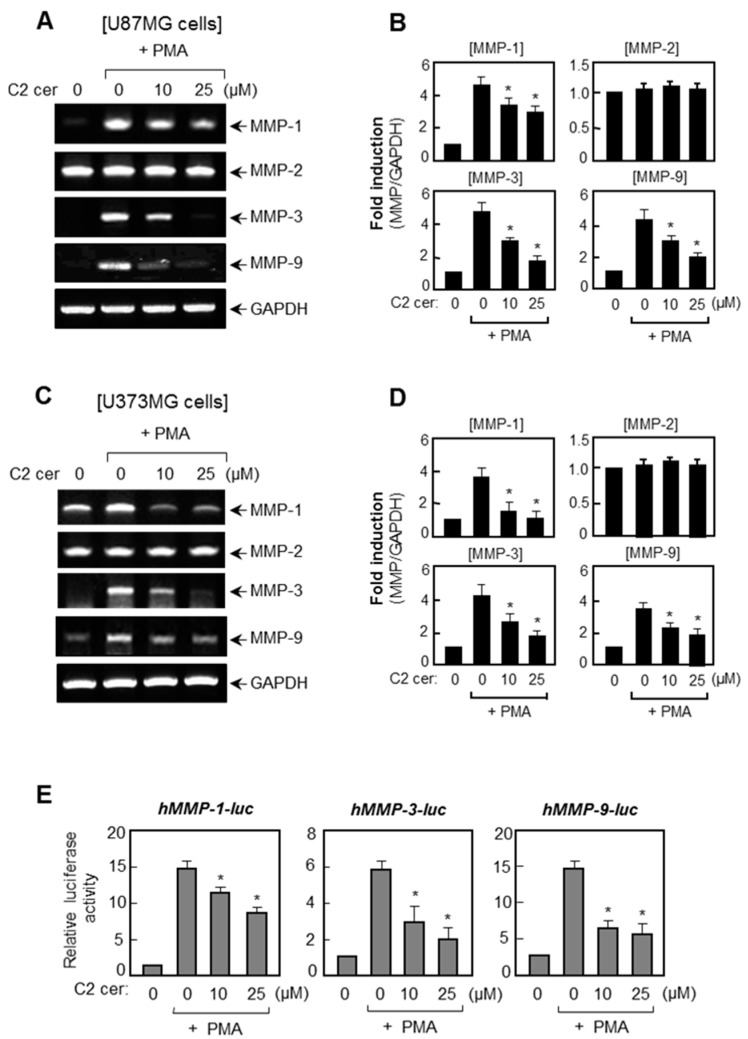
C2 ceramide suppresses the mRNA levels and promoter activities of *MMP-1, -3,* and *-9* in U87MG cells. (**A**–**D**) Cells were treated with C2 ceramide for 1 h before stimulation with phorbol myristate acetate (PMA) (50 ng/mL) for 6 h, and total RNA was isolated. Then, RT-PCR was performed to detect *MMPs* expressed from U87MG (**A**) and U373MG cells (**C**); Quantification of RT-PCR data are shown in the right panel (**B**; U87MG, **D**; U373MG cells); and (**E**) U87MG cells were transfected with *MMP-1*, *-3*, and *-9* reporter plasmids, and treated with C2 ceramide in the absence or presence of the PMA for 16 h. Then, cells were harvested and the luciferase assay was performed using the cell lysates. Data are the mean ± S.E.M. of three independent experiments. * *p* < 0.05, significantly different from PMA-treated samples.

Reverse transcription polymerase chain reaction (RT-PCR) was performed to investigate the effect of C2 ceramide on *MMP* expressions in PMA-stimulated U87MG and U373MG glioma cells. We found that PMA (50 ng/mL), which is a strong tumor inducer, significantly enhanced *MMP-1*, *-3*, and *-9* mRNA expressions, whereas pre-treatment with C2 ceramide resulted in an inhibition of the *MMP-1*, *-3*, and -*9* expressions in both the U87MG and U373MG cells (Figure 1A–D). However, *MMP-2* was constitutively expressed in glioma cells and PMA did not alter the expression level of *MMP-2* or C2 ceramide treatments. Moreover, C2 ceramide suppressed PMA-induced promoter activities of *MMP-1*, *-3*, and *-9* in U87MG cells (Figure 1E). Thus, the data indicates that C2 ceramide regulates *MMP-1*, *-3*, and *-9* at the transcriptional level. The concentration of C2 ceramide (up to 25 μM) used in these experiments did not affect the cell-viability (data not shown).

### 2.2. C2 Ceramide Inhibits the Protein Expressions of MMP-1, -3, and -9 in U87MG Glioma Cells

**Figure 2:**
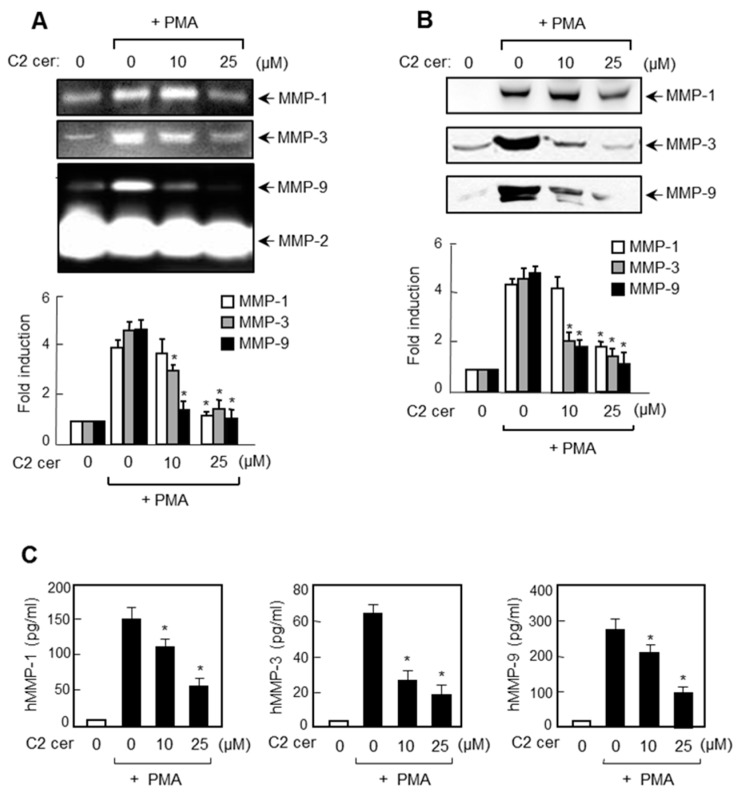
C2 ceramide inhibits the protein expressions of MMP-1, -3, and -9 in U87MG cells. (**A**) Cells were treated with C2 ceramide in the presence of PMA in serum free media for 24 h. The conditioned media were analyzed by gelatin zymography (detection of MMP-1, -2, and -9) and casein zymography (detection of MMP-3); (**B**) Western blot analysis for MMP expressions in the conditioned media using antibodies against MMP-1, -3 and -9. The fold inductions of each MMP protein level are shown in the bottom panel; and (**C**) U87MG glioma cells were treated with C2 ceramide in presence of PMA for 24 h. The levels of MMP-1, -3, and -9 secreted in the medium were analyzed through enzyme-linked immunosorbent assay (ELISA). Data correspond to the mean ± S.E.M. of three independent experiments. * *p* < 0.05; significantly different from the PMA treated sample.

We examined the effect of C2 ceramide on MMP secretion using gelatin and casein zymography. Gelatin zymography data showed that while markedly suppressing the PMA-induced secretion of MMP-1 and MMP-9, C2 ceramide did not affect MMP-2 (Figure 2A). In addition, casein zymography data showed that C2 ceramide inhibited MMP-3 secretion. Data from Western blot analysis suggest that the suppressed secretion of MMP-1, -3, and -9 was due to a decrease in the amount of the protein (Figure 2B). We further examined the effect of C2 ceramide on protein expression of MMPs using enzyme-linked immunosorbent assay (ELISA). As shown in Figure 2C, C2 ceramide significantly inhibited the protein expression of MMP-1, -3, and -9 in PMA-stimulated U87MG cells. Therefore, the data suggest that C2 ceramide is a broad spectrum inhibitor of MMP-1, -3, and -9 that plays a crucial role in glioma invasion.

### 2.3. C2 Ceramide Inhibits the in Vitro Invasion and Migration of U87MG Glioma Cells

**Figure 3:**
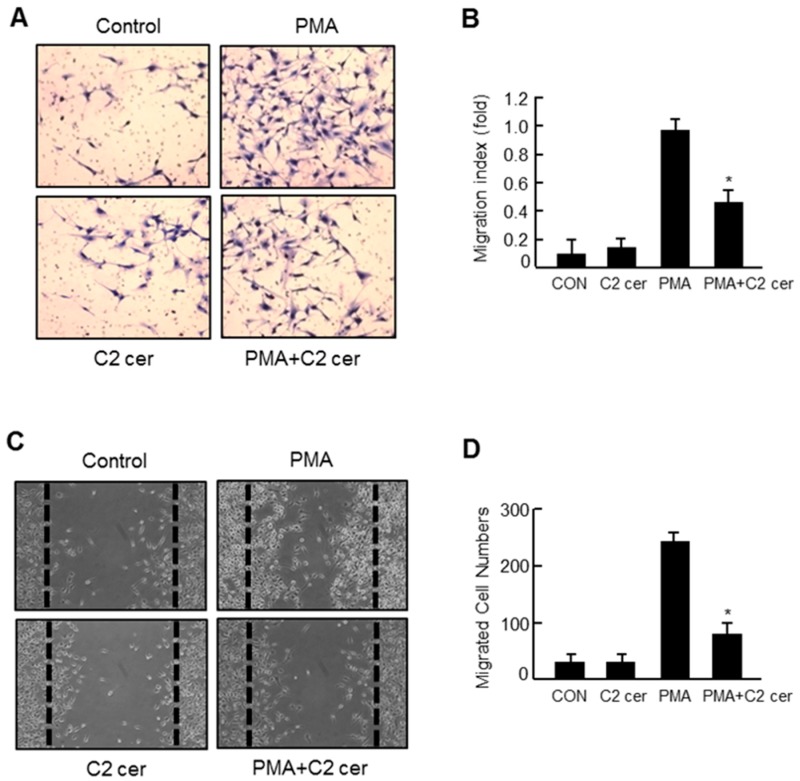
C2 ceramide inhibits the *in vitro* invasion and migration of U87MG glioma cells. (**A**) The effect of C2 ceramide (25 μM) on the invasion of U87MG cells was determined using the modified Boyden chamber method, as described in the Methods section. Cells were treated with C2 ceramide for 1 h before stimulation with PMA (50 ng/mL) for 24 h. Cells that invaded the lower surface of the membrane were fixed and stained; (**B**) cells were counted from at least 10 fields; data represent the mean ± S.E.M. of at least three independent experiments. * *p* < 0.05; significantly different from the PMA-treated sample; (**C**) the effect of C2 ceramide (25 μM) on the migration of U87MG cells was observed by wound healing assay as described in the Methods section; and (**D**) migratory cells were counted from four phase images at each point and then averaged for each experimental condition. *N* = 3, * *p* < 0.05; significantly different from the PMA-treated sample.

The effect of C2 ceramide on chemoinvasion was examined using Matrigel in U87MG glioma cells. We found that the PMA-induced invasion was significantly inhibited after a 24 h treatment with C2 ceramide without affecting cell viability (Figure 3A,B). In addition, C2 ceramide significantly inhibited the PMA-induced migration of U87MG cells as shown by the wound healing assay results (Figure 3C,D). Our group has previously reported that specific inhibitors of MMP-3 or -9 significantly inhibited the invasion in PMA-induced U87MG glioma cells [16]. Therefore, the inhibitory effect of C2 ceramide on *in vitro* invasion and migration might be related to the concomitant inhibition of MMP-1, -3, and -9 by C2 ceramide.

### 2.4. C2 Ceramide Inhibits DNA Binding and Promotor Activities of NF-κB and AP-1, Which Are Important Transcription Factors for MMP Gene Expression

**Figure 4:**
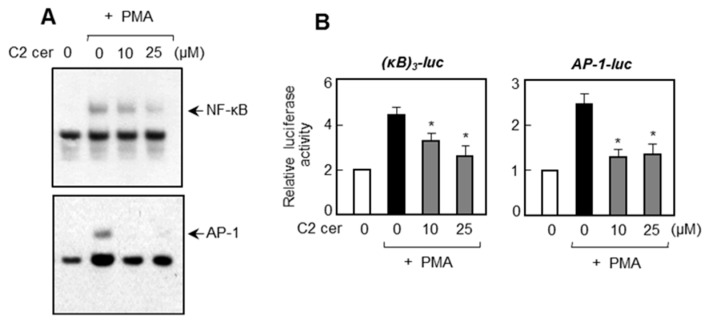
C2 ceramide inhibits DNA binding and transcriptional activities of NF-κB and AP-1. (**A**) Nuclear extracts were prepared from U87MG cells after treatment with PMA for 6 h in the absence or presence of C2 ceramide. Using the nuclear extracts, EMSA was performed to determine the effect of C2 ceramide on DNA binding activities of NF-κB and AP-1; and (**B**) U87MG cells were transfected with (κB)3-luc or AP-1-luc, and treated with C2 ceramide followed by PMA treatment. After 16 h, cells were harvested and luciferase assay was performed. Data correspond to the mean ± S.E.M. of three independent experiments. * *p* < 0.05; significantly different from the PMA treated samples.

Further mechanistic studies were investigated to examine the effects of C2 ceramide on NF-κB and AP-1, which are known to regulate *MMP* gene expression [17]. Electrophoretic mobility shift assay (EMSA) analysis showed that C2 ceramide inhibited the PMA-induced DNA binding activities of NF-κB and AP-1 in U87MG cells (Figure 4A). In addition, C2 ceramide inhibited the promoter activities of NF-κB and AP-1 as shown by reporter gene assays (Figure 4B). Therefore, the data suggest that the inhibition of NF-κB and AP-1 by C2 ceramide may, at least partly, contribute to the suppression of *MMP* gene expression.

### 2.5. C2 Ceramide Suppresses Mitogen-Activated Protein Kinase (MAPK) Phosphorylation and Reactive Oxygen Species (ROS) Production in PMA-Treated U87MG Glioma Cells

**Figure 5:**
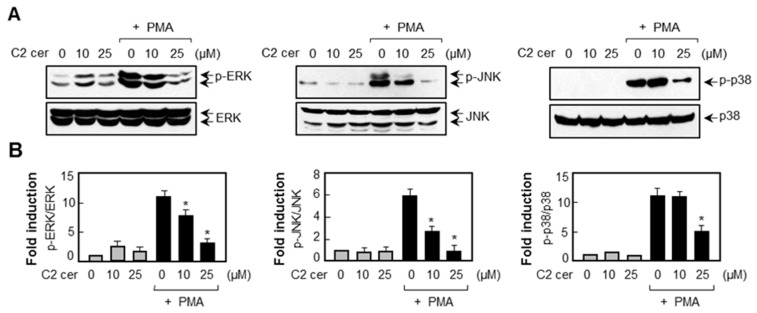
C2 ceramide suppresses PMA-induced phosphorylation of three types of MAPKs. (**A**) Cells extracts were prepared from U87MG cells treated with PMA for 30 min in the absence or presence of C2 ceramide and Western blot analysis was performed using antibodies against phosphorylated or the total forms of MAPKs (ERK, JNK and p38 MAPK); (**B**) quantification of Western blot data. Levels of the active forms of MAPKs were normalized with respect to the total form and expressed as relative fold changes in comparison to control samples, which were arbitrarily set to 1.0. Values correspond to the mean ± S.E.M. of three independent experiments. * *p* < 0.05; significantly different from the PMA-treated sample.

**Figure 6:**
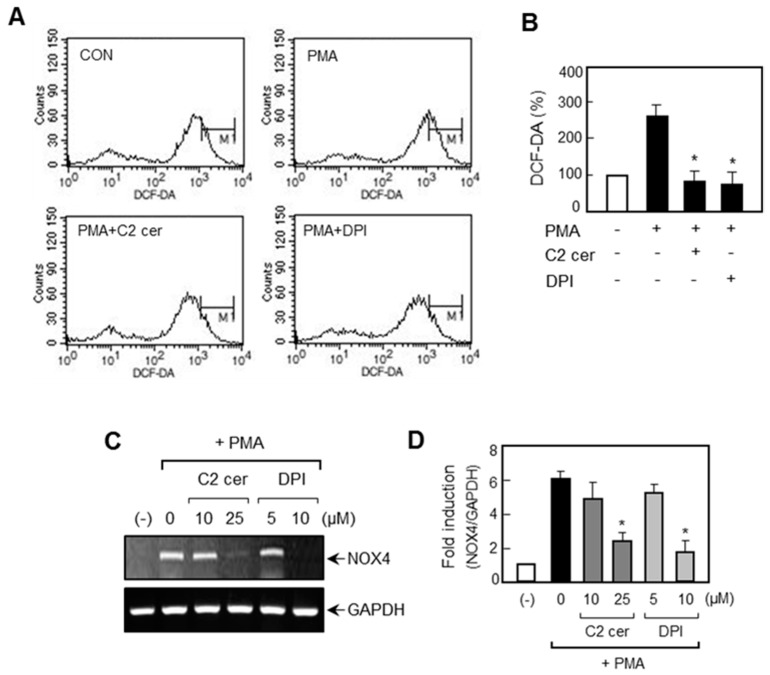
C2 ceramide suppresses ROS production via inhibition of *NOX4* in PMA-treated U87MG cells. (**A**) Cells were treated with C2 ceramide or DPI followed by PMA treatment for 1 h, and the level of intracellular ROS production was evaluated by flow cytometry analysis; (**B**) level of ROS production was quantified using a densitometer. * *p* < 0.05; significantly different from the PMA-treated sample; (**C**) RT-PCR was performed to determine the effect of C2 ceramide or DPI on *NOX4* mRNA expression in PMA-treated U87MG cells; and (**D**) quantification of RT-PCR data. * *p* < 0.05; significantly different from the PMA-treated sample.

Next, we examined the effects of C2 ceramide on MAP kinases and ROS, which are upstream signaling regulators of MMP expression [18,19]. Western blot analysis showed that C2 ceramide inhibited the phosphorylation of ERK, JNK, and p38 MAPK in PMA-stimulated U87MG cells (Figure 5). Furthermore, flow cytometry analysis revealed that C2 ceramide inhibited ROS production induced by PMA (Figure 6A,B). Next we examined the effect of C2 ceramide on NADPH oxidase, the major enzyme involved in ROS generation. Our preliminary data showed that among the five types of NADPH oxidase (NOX1-5), only NOX4 was induced by PMA in U87MG glioma cells (data not shown). In the present study, we found that C2 ceramide inhibited *NOX4* mRNA expression in PMA-stimulated U87MG cells (Figure 6C,D).

### 2.6. Treatment of ROS Inhibitor Mimicked the Effects of C2 Ceramide on MMP Gene Expression and NF-κB/AP-1 via Inhibition of p38 MAPK

**Figure 7:**
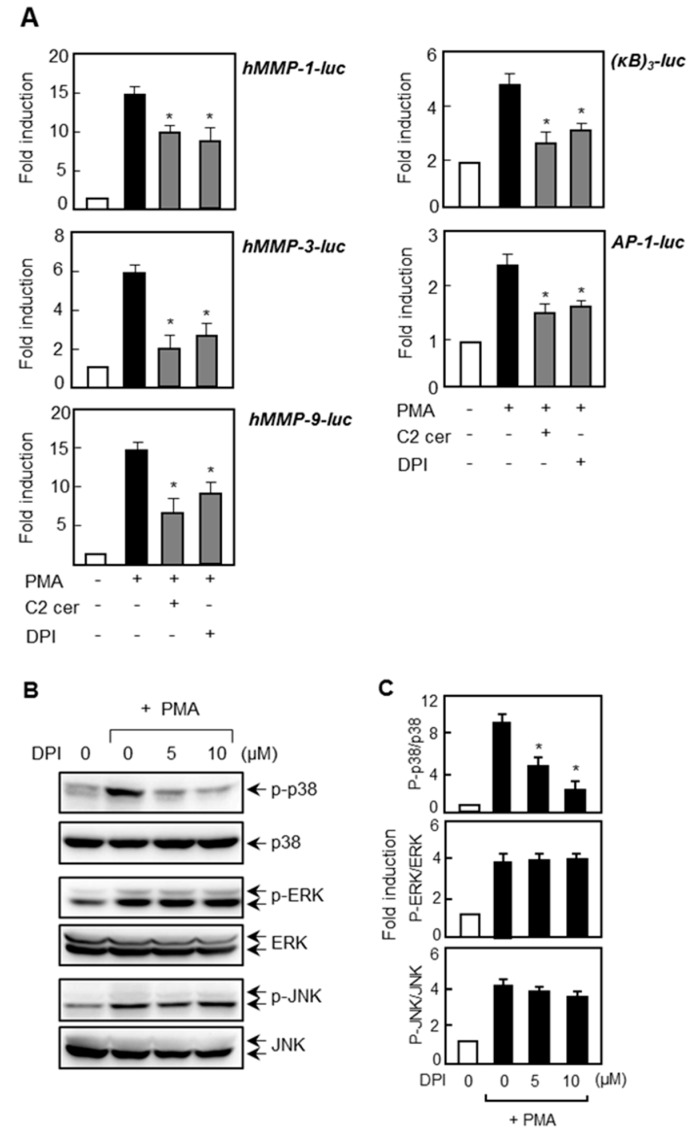
Effect of ROS inhibitor on the expression of *MMP*s and MAPK phosphorylation. (**A**) U87MG cells were transfected with reporter plasmids of *MMP*s (*MMP-1, -3*, and -*9*), NF-κB, and AP-1. Cells were pretreated with C2 ceramide or DPI for 1 h before being treated with PMA. After 6 h, cells were harvested and luciferase assay was performed; (**B**) cells were treated with DPI followed by PMA treatment for 30 min, and the phosphorylated and total form of MAPKs were detected using Western blot; and (**C**) quantification of Western blot data. * *p* < 0.05; significantly different from the PMA-treated sample.

To investigate the possible involvement of ROS in *MMP* gene expression, we examined the effect of NADPH oxidase inhibitor, DPI, on the promoter activities of *MMP-1*, *-3*, and *-9*. As shown in Figure 7A, treatment with DPI inhibited the transcriptional activities of three types of MMPs. Moreover, DPI inhibited the NF-κB and AP-1 reporter gene activities. When we examined the effect of DPI on MAPKs, we observed that DPI inhibited the PMA-induced phosphorylation of p38 MAPK without affecting ERK or JNK (Figure 7B,C). The data collectively suggest that ROS-p38 MAPK signaling plays an important role in *MMP* gene expressions by modulating NF-κB and AP-1. On the other hand, ERK and JNK control *MMP* gene expression independently of ROS production.

## 3. Discussion

In the present study, we demonstrate that C2 ceramide inhibits MMP-1, -3, and -9 expressions in PMA-stimulated human astroglioma cells. We also observed that the invasion and migration of glioma cells were significantly inhibited by C2 ceramide by performing Matrigel invasion assay and wound healing assay. Further mechanistic studies revealed that ROS production and MAPK signaling pathways are involved in C2 ceramide-mediated MMP regulation in PMA-stimulated U87MG glioma cells.

ROS, including the superoxide anion, hydrogen peroxide, and the hydroxyl radical, serve as regulators or secondary messengers of signal transduction pathways for cell proliferation, survival, and apoptosis [20]. Previous studies reported that ROS play essential roles in neoplastic proliferation and angiogenesis by inducing growth factors [19,20,21]. In addition, ROS is known to mediate *MMP* gene expression [19]. Hydrogen peroxide activates Ras and MAPK signaling pathways, inducing the expression of MMPs responsible for the migration of cancer cells [22,23,24]. The MMP-1 level can be redox dependent and activated by Ets-1 and c-Jun via ERK and JNK signaling pathways [25]. Similarly, TGF-β1 directly activates MMP-9 expression through ROS-dependent ERK and NF-κB pathways in vascular smooth muscle cells [26]. In the present study, we found elevated intracellular ROS levels in PMA-treated U87MG cells and observed the suppression of *MMP* gene expression by C2 ceramide through the inhibition of the ROS-p38 MAPK-NF-κB/AP1 signaling axis.

NOX family NADPH oxidase is the major player in ROS generation mechanisms and has pivotal roles in various physiological and pathological processes [19,27,28]. In phagocytic cells, the NADPH oxidase consists of the catalytic *gp91phox* and *p22phox*, cytosolic components including *p47phox*, *p67phox*, and the small Rho guanosine triphosphatase (GTPase) Rac1. Several homologs of *gp91phox* (*NOX2*), which are *NOX1*, *NOX3*, *NOX4*, and *NOX5*, have been identified in non-phagocyticcells [27,29,30]. It was previously reported that *NOX4* is prominently expressed in various neuroepithelial tumors, and enhanced expression of *NOX4* appears to be involved in cell proliferation and survival in glioma cells [31]. In accordance with this, we found that *NOX4* is expressed at higher level than other NOX proteins in PMA-treated U87MG cells (data not shown). The NOX4-mediated ROS are known to contribute to cycling hypoxia-promoted tumor progression with activation of HIF-1α in glioblastoma cells and xenografts [32]. Furthermore, NOX4-generated ROS are required for the cycling hypoxia-induced glioma invasion and infiltration through the activation of ERK- and NF-κB-mediated *MMP-9* expression [33]. In the present study, we showed that C2 ceramide inhibits PMA-induced *NOX4* expression, and the inhibition of *NOX4* by DPI significantly suppressed the expression of *MMP-1*, *-3*, *-9*, as well as its upstream NF-κB/AP-1 and p38 MAPK activities. The data suggests that NOX4-mediated ROS generation may be a potential target towards the control of *MMP* expression and glioma invasion.

**Figure 8:**
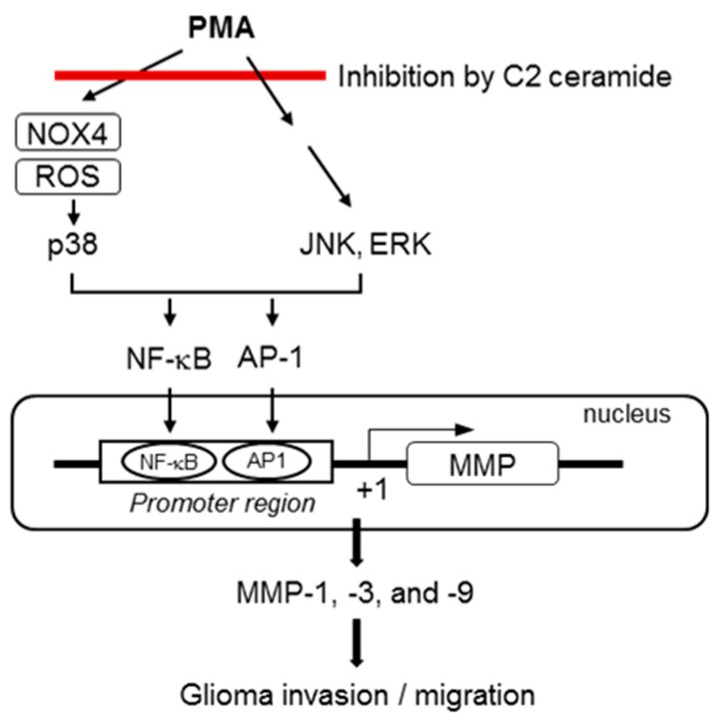
Proposed signaling pathways for C2 ceramide-mediated inhibition of invasion and migration of U87MG glioma cells.

Previous studies carried out by our group have reported that inhibition of MAPKs attenuated glioma invasiveness and *MMP* gene expression [18,34]. In addition, the MAPK-specific inhibitors suppressed NF-κB and/or AP1 activities in PMA-stimulated U87MG cells [35]. In this study, we found that C2 ceramide inhibits the phosphorylation of three types of MAP kinases. Among them, p38 MAPK was shown to be governed by ROS signaling, whereas ERK or JNK were not affected by ROS. Therefore, the data collectively suggest that the ROS-p38 MAPK pathway plays an important role in *MMP* gene expression by modulating NF-κB/AP-1, while ERK and JNK control *MMP* gene expression independently of ROS production (Figure 8).

## 4. Materials and Methods

### 4.1. Reagents

C2 ceramide was obtained from BIOMOL International (Plymouth Meeting, Kelayres, PA, USA). All reagents used for cell culture containing penicillin/streptomycin, trypsin, and Dulbecco/Vogt modified Eagle’s minimal essential medium (DMEM) were obtained from Gibco BRL (Grand Island, NY, USA). Antibodies against the phospho-/total form of p38 MAPK, extracellular signal-regulated kinase (ERK) 1/2, and stress-activated protein kinases (SAPK)/Jun amino-terminal kinases (JNK) were purchased form Cell Signaling Technology (Danvers, MA, USA). Antibodies against MMP-1, -3, and -9 were purchased from Abcam (Cambridge, UK). All other chemicals were purchased from Sigma-Aldrich (St. Louis, MO, USA), unless stated otherwise.

### 4.2. Cell Culture and Transient Transfection Assays

Human astroglioma U87MG and U373MG cells (American Type Culture Collection, Manassas, VA, USA) were grown in DMEM, supplemented with 10% fetal bovine serum (Hyclone, South Logan, UT, USA), streptomycin, and penicillin. Transfection was performed by using the Convoy reagent (ACTGene, Inc., Piscataway, NJ, USA). MMP reporter plasmids (hMMP1-luc, hMMP3-luc, and hMMP9-luc) were constructed by our group [18]. NF-κB and AP-1 reporter plasmids were purchased from Clontech (Mountain View, CA, USA). After 48 h of transfection of the reporter plasmids, cells were harvested and luciferase assays were performed as previously described [18].

### 4.3. RT-PCR

Total RNA (1 μg) was extracted from appropriately treated glioma cell lines and reverse-transcribed in a reaction mixture containing 1 U RNase inhibitor, 500 ng random primers, 3 mM MgCl_2_, 0.5 mM dNTP, 1 X RT buffer, and 10 U reverse transcriptase (Promega, Madison, WI, USA). The synthesized cDNA was used as a template for the PCR. RT-PCR was performed using GoTaq polymerase (Promega) and primers, as shown in Table 1.

### 4.4. Zymography and Enzyme-Linked Immunosorbent Assay (ELISA)

Activity of gelatinase subfamily of MMP-1 and MMP-9 secreted in conditioned media were assayed using gelatin-substrate gel electrophoresis, as described in a previous study [35]. Briefly, conditioned media were concentrated by precipitation with two volumes of absolute ethanol, re-suspended with a 1× sample buffer without reducing agent, and then added to a 7.5% SDS-PAGE gel containing 0.1% (*w*/*v*) gelatin. After electrophoresis at 4 °C, the gel was washed briefly with water and then 2.5% Triton X-100 (*v*/*v*) for 30 min at RT to remove SDS, allowing the protein to renature. The protein was then subsequently incubated in a substrate buffer (50 mM Tris-HCl, pH 7.5, 1 mM ZnCl_2_, and 5 mM CaCl_2_) at 37 °C for 48–72 h. After staining and destaining of the gel, a zymogram was obtained. The casein lytic activity of MMP-3 in conditioned media was determined using 12% zymogram casein gels (Invitrogen, Carlsbad, CA, USA). The other conditions (running of the gel, renaturation and staining) were the same as with gelatin zymography. The levels of MMP proteins in the medium were determined using human MMP ELISA kits (RayBiotech, Inc., Norcross, GA, USA), following the manufacturer’s instructions.

### 4.5. Matrigel Invasion Assay

Invasion assays were carried out using modified Boyden chambers as described previously [36]. Cells were plated on the Matrigel-coated Transwell with or without C2 ceramide in the presence of PMA. The medium in the lower chambers also contained 0.1 mg/mL bovine serum albumin. The inserts were incubated at 37 °C for 24 h. Non-invading cells were removed by wiping the upper surface of the membrane, and the invading cells were fixed with methanol and stained with hematoxylin. Randomly selected fields were counted under a light microscope.

### 4.6. Wound Healing Assay

U87MG cells were cultured to confluence in six-well culture plates for 24 h. The monolayer cells were wounded by manually scratching the surface with a sterile 200-μL pipette tip to create definite scratches in the center of the clear field dishes. Then, the cells were incubated with serum-free DMEM for 1 h. Subsequently, cells were pretreated with C2 ceramide for 1 h prior to treatment with PMA. Cells migrating from the leading edge were finally photographed after 24 h. Migratory cells were enumerated from the resulting four phase images taken at each distinct point and averaged for each experimental condition.

### 4.7. Western Blot Analysis

Cells were treated with C2 ceramide in the absence or presence of PMA and total cell lysates were prepared as described previously [35]. The proteins (20–100 μg) were heated within a 4× SDS sample buffer and separated by SDS-PAGE gel electrophoresis and transferred to nitrocellulose membranes (GE Healthcare, Chalfont, St. Giles, Buckinghamshire, UK). The membranes were blocked with 5% bovine serum albumin in 10 mM Tris-HCl containing 150 mM NaCl and 0.5% Tween-20 (TBST) and then incubated with primary antibodies (1:1000) against the phospho- or the total forms of MAP kinases. After TBST washing, horseradish peroxidase-conjugated secondary antibodies (1:2000 dilution in TBST; New England Biolabs, Ipswich, MA, USA) were applied and the blots were developed using an enhanced chemiluminescence detection kit (Thermo Fisher Scientific, Waltham, MA, USA).

### 4.8. Electrophoretic Mobility Shift Assay (EMSA)

U87MG cells were treated with PMA in the absence or presence of C2 ceramide, and nuclear extracts were prepared as described in a previous study [16]. Nuclear proteins (5 μg) were incubated with ^32^P-labeled AP-1 or NF-κB probe on ice for 30 min, and the DNA-protein complex was resolved on a 5% acrylamide gel and visualized by autoradiography. The oligonucleotides containing consensus sequences of the AP-1 or NF-κB were purchased from Promega.

### 4.9. Flow Cytometry Analysis

The production of ROS was monitored by flow cytometry using H_2_DCF-diacetate (DCF-DA). U87MG cells were treated with C2 ceramide for 1 h, followed by PMA stimulation for an additional 1 h. Subsequently, the cells were washed twice with PBS to remove the extracellular compounds, before DCF-DA (50 μM) was added and excited using an argon laser. Fluorescence was then detected through a 525-nm (FL1-H) band-pass filter using a flow cytometer (BD FACSCalibur, Franklin Lakes, NJ, USA).

### 4.10. Statistical Analysis

Unless otherwise stated, all experiments were performed in triplicate, each repeated at least three times. The data are presented as mean ± S.E.M. and statistical comparisons between groups were performed by using one-way analysis of variance, followed by Newman–Keuls test. A *p* value <0.05 was considered significant.

## 5. Conclusions

Through this study, we report for the first time that C2 ceramide inhibits glioma invasion/migration by inhibiting *MMP* gene expression. ROS and MAPK signals were shown to be involved in C2 ceramide-mediated inhibition of MMPs. Therefore, our data collectively suggest that C2 ceramide may be a potential therapeutic agent for malignant glioma.

## Figures and Tables

**Table 1:** Primers used in PCR reactions.

Gene	Forward Primer (5′→3′)	Reverse Primer (5′→3′)	Size
*MMP-1*	ATATCGGGGCTTTGATGTACC	AGCTGTAGATGTCCTTGGGGT	408 bp
*MMP-2*	GAAGTATGGGAACGCCGATGG	TTGTCGCGGTCGTAGTCCTCA	311 bp
*MMP-3*	GATATAAATGGCATTCAGTCCCTC	TCCTTGCTAGTAACTTCATATGCG	287 bp
*MMP-9*	ATGT ACCCTATGTACCGCTTCACT	CAGAGAAGAAGAAAAGCTTCTTGG	496 bp
*GAPDH*	GGTCGGTGTGAACGGATTTGGCCG	GGTTCACACCCATCACAAACATGG	395 bp

## Abbreviations

AP	activator protein
DCF	dichlorodihydrofluorescein
DMEM	Dulbecco/Vogt modified Eagle’s minimal essential medium
DPI	diphenylene iodonium
ECM	extracellular matrix
ELISA	enzyme linked immunosorbent assay
EMSA	electrophoretic mobility shift assay
ERK	extracellular signal-regulated kinase
JNK	c-Jun N-terminal kinase
MAPK	mitogen-activated protein kinase
MMP	matrix metalloproteinase
NF-κB	nuclear factor-κB
PMA	phorbol myristate acetate
ROS	reactive oxygen species
